# Unveiling the genomic potential of *Pseudomonas* type strains for discovering new natural products

**DOI:** 10.1099/mgen.0.000758

**Published:** 2022-02-23

**Authors:** Zaki Saati-Santamaría, Nelly Selem-Mojica, Ezequiel Peral-Aranega, Raúl Rivas, Paula García-Fraile

**Affiliations:** ^1^​ Microbiology and Genetics Department, University of Salamanca, 37007 Salamanca, Spain; ^2^​ Institute for Agribiotechnology Research (CIALE), 37185 Salamanca, Spain; ^3^​ Centro de Ciencias Matemáticas UNAM, 58089, Morelia, Michoacán, Mexico; ^4^​ Associated Research Unit of Plant-Microorganism Interaction, University of Salamanca-IRNASA-CSIC, 37008 Salamanca, Spain

**Keywords:** BGCs, BiG-SCAPE / CORASON, comparative genomics, EvoMining, pan-genome, secondary metabolites

## Abstract

Microbes host a huge variety of biosynthetic gene clusters that produce an immeasurable array of secondary metabolites with many different biological activities such as antimicrobial, anticarcinogenic and antiviral. Despite the complex task of isolating and characterizing novel natural products, microbial genomic strategies can be useful for carrying out these types of studies. However, although genomic-based research on secondary metabolism is on the increase, there is still a lack of reports focusing specifically on the genus *Pseudomonas *. In this work, we aimed (i) to unveil the main biosynthetic systems related to secondary metabolism in *Pseudomonas* type strains*,* (ii) to study the evolutionary processes that drive the diversification of their coding regions and (iii) to select *Pseudomonas* strains showing promising results in the search for useful natural products. We performed a comparative genomic study on 194 *Pseudomonas* species, paying special attention to the evolution and distribution of different classes of biosynthetic gene clusters and the coding features of antimicrobial peptides. Using EvoMining, a bioinformatic approach for studying evolutionary processes related to secondary metabolism, we sought to decipher the protein expansion of enzymes related to the lipid metabolism, which may have evolved toward the biosynthesis of novel secondary metabolites in *Pseudomonas *. The types of metabolites encoded in *Pseudomonas* type strains were predominantly non-ribosomal peptide synthetases, bacteriocins, N-acetylglutaminylglutamine amides and ß-lactones. Also, the evolution of genes related to secondary metabolites was found to coincide with *Pseudomonas* species diversification. Interestingly, only a few *Pseudomonas* species encode polyketide synthases, which are related to the lipid metabolism broadly distributed among bacteria. Thus, our EvoMining-based search may help to discover new types of secondary metabolite gene clusters in which lipid-related enzymes are involved. This work provides information about uncharacterized metabolites produced by *Pseudomonas* type strains, whose gene clusters have evolved in a species-specific way. Our results provide novel insight into the secondary metabolism of *Pseudomonas* and will serve as a basis for the prioritization of the isolated strains. This article contains data hosted by Microreact.

## Data Summary

Big tables and special files can be found in Zenodo, under the following link http://sci-hub.tw/10.5281/zenodo.4539927

In there, the following objects are included:

- Data S1: A spreadsheet with four tables: (Table S1) Accession numbers and genome characteristics of the genomes used in this study; (Table S2) summary of BGCs predicted for each of the genomes; (Table S3) proteins used for the conformation of the CentralDatabase for Evomining; (Table S4) AMP predictions.
- Data S2: A folder with all the .gbk files for the BGCs predicted by antismash. These files were used as input for BiG-SCAPE.
- Data S3: Different trees summarizing the distribution of BGCs in the *Pseudomonas* genus.
- Data S4: A file for the visualization of the BGCs-SSN in Cytoscape.
- Data S5: Different trees and tanglegrams in which the evolution of GCFs is depicted.
- Data S6: A file for the visualization of the AMPs-SSN in Cytoscape.

Evomining trees and metadata have been deposited in Microreact through the following links:

(1) https://microreact.org/project/a6j19Z7rVWLQbNkgJjAAkv;
(2) https://microreact.org/project/jeB8iymmVuqjocTnitFxvL;
(3) https://microreact.org/project/jAXhaH5kiCKCeNfrYiwNVU;
(4) https://microreact.org/project/29fHRUP6h5enzX2ruK5eTC;
(5) https://microreact.org/project/rR9Dqzg7uFbKsyLsHakG9M;
(6) https://microreact.org/project/3S5zZe3NbMoGwHXHfZDbVF;
(7) https://microreact.org/project/4CcJmrcRzfTU1NneKYprgd;
(8) https://microreact.org/project/2kKgaubCTXpFx7hqGosM4v;
(9) https://microreact.org/project/eqWSWgLfS3TZm7eosF34pA;
(10) https://microreact.org/project/wtWzxgL2uKbG97FvgFzR2A;
(11) https://microreact.org/project/oQvmzS9WrThsAKKFZNzYPZ;
(12) https://microreact.org/project/35KUWdaoun3PQMyNeGJuwT;
(13) https://microreact.org/project/5gdXTELHJwZYTAfHgiSwBS;
(14) https://microreact.org/project/ftwXpDCF15hh91ctCy3YbE;
(15) https://microreact.org/project/vXLU1bGcx5dFZgE9sz6zgL;
(16) https://microreact.org/project/2y4wmrMytAAdnW2irQpbtt;
(17) https://microreact.org/project/bbYpqhxAvyLLaFF6Uy1MH1;
(18) https://microreact.org/project/d8w6z7pbH41R7P5WQ9Y6Dn;
(20) https://microreactorg/project/9iGvZpDzYf3DVVmyBQvn.

The authors confirm all supporting data, code and protocols have been provided within the article or through supplementary data files.

**Impact Statement**

Genomics is a useful field for discovering undescribed secondary metabolites in bacteria, which may have the potential to become new drugs or have ecological and biotechnological relevance. In this work we aimed to examine the genetic potential of different *Pseudomonas* species to produce secondary metabolites, and in particular to identify those that have not been previously described. We have determined the most common biosynthetic systems of secondary metabolites within the selected genomes, as well as their evolutionary dynamics, and have found that the evolution of these gene clusters matches the evolution of the *Pseudomonas* species. We also suggest key enzymes that may be implicated in novel lipid-related biosynthetic systems, and have detected potential antimicrobial peptides encoded by these genomes. Based on this research, several type strains have been selected based on their potential for producing novel natural products. Finally, this work will allow us to more effectively select and prioritize biosynthetic gene clusters for the discovery of new bioactive metabolites.

## Introduction

Microbes synthesize an immense variety of natural products that aid in their competition and survival in diverse environments. Over the last century, microbes have been studied with the intent to discover novel and valuable secondary metabolites, and although hundreds have already been applied in clinical settings and for industrial purposes, such as antibiotics and antitumor molecules [1], many diseases still lack effective treatments. Also, current problems, such as that of antimicrobial resistance and the ongoing COVID-19 pandemic, have highlighted the need for identifying effective compounds. Thus, the isolation and characterization of novel microbial metabolites are greatly important, as these compounds can potentially help to overcome such threats [2]. Still, in the past few decades, the high rate of re-discoverability of secondary metabolites has supposed a slowdown in the characterization of novel molecules [3].

Nowadays, the cost of genome sequencing is much more accessible, owing to the latest advances in high-throughput DNA sequencing. Consequently, the field of genomics is a promising area that guides the search for natural products [2]. Also, the study of genomes has allowed a deeper understanding of unknown metabolites encoded by cryptic Biosynthetic Gene Clusters (BGCs) in bacteria, and fungi and the development of bioinformatic tools for sequence analysis has facilitated the exploration for genes related to secondary metabolism [4–7]. The combination of genomic analyses with phylogenomics and comparative genomics has allowed the identification of certain promising strains for drug discovery [8, 9]. Moreover, the creation of databases holding information on already described BGCs related to the production of specialized metabolites has allowed comparisons to be made and novel BGCs to be prioritized for research purposes [10]. Additionally, there are new concepts regarding the analyses of the microbial genomes that have emerged as promising ways to discover new types of secondary metabolites. For example, the EvoMining approach uses evolutionary principles to identify enzyme expansions (based on gene duplications) implicated in novel biosynthetic systems [11]. The authors argue that secondary metabolite enzymes have evolved from central or primary metabolism through enzyme expansion, where enzymes are recruited to carry out new functions and have attempted to try to identify them through the use of protein phylogenies. Thus, the study of duplicated genes/proteins encoding a certain enzyme can lead to the discovery of new BGCs [12–15].

One of the main advantages of the analysis of the genomes is that it allows novel metabolites to be searched for in a guided fashion. Once a BGC has been selected for subsequent analysis, its expression may be modulated by its genetic manipulation allowing researchers to more easily activate cryptic BGCs [16]. Thus, genome-guided approaches may be more profitable than high-throughput screenings of microbes, in which the expression of BGCs are conditioned by culture conditions and the composition of the culture media selected, and for which the high re-discovery rate supposes the main disadvantage [3]. In this sense, mining genomes may allow finding cryptic uncharacterized BGCs and to discover its encoded metabolite with lower re-discovery risk [16].

*Pseudomonas* is one of the most diverse and ubiquitous bacterial genera in nature. It is commonly found associated with animals, plants, soil, water and many other ecological niches, where these organisms can endure highly diverse environmental conditions [17–20]. To survive and compete in these micro-habitats, *Pseudomonas* strains can produce a repertoire of secondary metabolites, which protect them from extreme conditions or aid in the fight against other microbial competitors in the same niche [17, 21, 22]. Many secondary metabolites have been discovered from *Pseudomonas* strains, and some of the most studied include pyoverdines, pyocyanins and 2,4-diacetylphloroglucinol [23, 24]. Indeed, the genetic background for many of these metabolites has been experimentally elucidated, and, so far, 68 *Pseudomonas* BGCs have been deposited in the MIBIG database [23, 25, 26].

Comparative genomics is aiding in the study of the secondary metabolism related to the biosynthetic machinery in different bacterial taxa [27–29]. These studies have uncovered an abundance of diverse natural products and their corresponding BGCs. Regarding *Pseudomonas* bacteria, Gutiérrez-García *et al*. [30] carried out a phylogenomic analysis in which evidence was found supporting the correlation between the evolution of six *Pseudomonas* species and the chemical diversity of 2,4-diacetylphloroglucinol (DAPG). However, to date, no general assessment has been carried out involving the genetic evolution and distribution of the genetic background of secondary metabolites in the genus *Pseudomonas *.

A bacterial type strain is the taxonomic representative for a certain taxon. Type strains are used for species description (i.e. phylogenies are based on their genes, genome similitude indexes are calculated with the genomes of this strains, phenotypic and chemotaxonomic features are investigated on these, etc.) and should be mandatory available in public bacterial collections [31, 32]. Thus, studying these strains instead of private strains is beneficial for science reproducibility and may help researchers to continue previous research on them. Moreover, the correct taxonomy of these strains is more trustworthy than the species assignment for other strains. Hence, in this study, we provide a holistic examination of the diversity and evolution of genes related to secondary metabolism in *Pseudomonas *. All available genomes from *Pseudomonas* type strains were used to predict the metabolic pathways involved in this type of metabolism. Our analyses have revealed the biosynthetic diversity of the type strains of this genus, and have shed light on the evolution of natural products produced by *Pseudomonas *. Interestingly, we found that one of the most abundant types of BGCs in nature, polyketide synthases (PKS), is not broadly represented in this genus. PKS are enzymes that build secondary metabolites by assembling lipid-related molecules, mainly acyl CoA monomers [33]. Thus, we employed evolutionary-based approaches for deciphering the implications of lipid or fatty acid metabolism in the secondary metabolism of *Pseudomonas *. Our work has sought to identify and evaluate novel types of BGC and their potential to produce new valuable secondary metabolites. This study may serve as the basis for the prioritization of BGCs useful for drug development, as well as *Pseudomonas* type strains that are publicly available in micro-organism collections and available for this area of research.

## Methods

### Genome download and taxa curation

All genomes available in NCBI (June 2020) obtained from type strains of the genus *Pseudomonas* were downloaded. All strains were evaluated using the List of Prokaryotic names with Standing in Nomenclature (LPSN, https://lpsn.dsmz.de/) to identify only the validated species from *Pseudomonas *. Genomes obtained from type strains from the same species were removed to preserve one genome per species. In total, 194 genomes from different *Pseudomonas* species (type strains) were selected for further analyses (Data S1: Table S1, available with the online version of this article). These strains were selected because type strains are available to researchers in culture collections and have been unambiguously assigned to a taxon.

### Gene calling, genome annotation and quality assessment

To prevent annotation biases, we avoided already published annotations and we performed the same processes to the genomes of this work. All 194 genomes were annotated using rast (v2.0) [34] in batch mode. Public scripts were employed (https://github.com/nselem/myrast) for the multiple submission of contigs and for retrieving the annotated genomes.

Genomes were evaluated using quast (v5.0.2) [35] and busco (v4.0.6) [36] to ascertain the quality and completeness of each genome.

### Analysis of the diversity of encoded secondary metabolites

To predict and analyse BGCs related to secondary metabolism, the genomes were annotated using antiSMASH (v5.1.2) [4] This programme was executed locally through full-featured runs on six CPUs. The pairwise distance among predicted BGCs and sequence similarity networks (SSNs) were performed using the BiG-SCAPE (Biosynthetic Gene Similarity Clustering and Prospecting Engine) tool [6] (v1.0.1), on ‘auto’ mode, by comparing our predicted BGCs against those BGCs available in the MIBiG database (v1.4) [37] and by combining all the BGC classes into a single network. Multi-locus phylogenies of gene cluster families (GCFs) were constructed using the CORASON (CORe Analysis of Syntenic Orthologs to prioritize Natural Product Biosynthetic Gene Clusters) tool implemented in the BiG-SCAPE workflow [6]. The input for this programme comprised the GenBank files of each of the BGCs predicted by antiSMASH. The output SSN was visualized using Cytoscape (v3.7.2) and the BGC annotations generated by BiG-SCAPE were added to Cytoscape networks. Annotations for MIBiG BGCs were made according to the MIBiG database.

Heatmaps, scatterplots and histograms were performed with the ggplot2 package (v3.3.2) in R (v4.0.2) [38].

### Analysis of secondary metabolites derived from lipid metabolism

To investigate the possible evolution of lipid functions concerning to the biosynthesis of novel secondary metabolites, we examined through the use of EvoMining the gene expansions recruited in the biosynthesis of secondary metabolites [11]. To this end, a pan-genome analysis was carried out using Proteinortho on all 194 genomes selected (v6.0.16) [39]. Clusters of orthologous proteins were generated considering an amino acid similarity threshold of 70% over the amino acid FASTA files derived from the rast gene calling. Then, proteins encoded by the type strain of the genus, *P. aeruginosa* DSM 50071^T^ were annotated using eggNOG-mapper (v5) [40]. All of the *P. aeruginosa* DSM 50071^T^ proteins annotated within the COG functional category ‘lipid transport and metabolism’ were pre-selected. Then, all of the proteins belonging to the orthologous groups with >194 copies within the pan-genome were finally selected. Subsequently, a custom central-metabolism database for EvoMining, which we called CENTRALlipid_DB, was assembled. The genome database was conformed with the 194 rast annotated genomes in the amino acid FASTA format and the files (.gbk) of the BGC previously predicted using antiSMASH were used for conforming the antiSMASH database for EvoMining with the antiSMASH_DB.pl script. A heatmap based on the number of orthologous copies for each of the proteins in the CENTRALlipid_DB was created. For this, data were standardized as *z*-scores and the values were linearly mapped using rgb colours; the lower values are purple, and the higher ones are represented as yellow squares. Output trees were visualized in Microreact [41] by uploading their .tsv (annotation) and .nwk (tree structure) files.

### Phylogeny and tree annotation

A phylogenomic tree representing the 194 genomic assemblies of this analysis was constructed using ubgc (Up-to-date Bacterial Core Gene) (v3.0) [42]. This programme extracts, concatenates and aligns up to 92 housekeeping genes before drawing the tree. Default settings were used. The Interactive Tree Of Life (iTOL) tool (v4) [43] was employed to display and annotate the phylogenetic tree of the genome data and the predicted BGC for each genome.

Diverse phylogenetic trees were created with the different species contained in each of the gene cluster Families (GCFs) trees generated with the BiG-SCAPE workflow. Both trees were compared using the tanglegrams generated with Dendroscope (v3.7.2) [44].

### Antimicrobial peptides (AMPs)

AMPs were predicted from the genome contigs using the programme macrel (Meta[genomic] AMPs Classification and REtrievaL) (v0.4.0) [45], locally installed. The predicted AMPs sequences were converted to an amino acid FASTA file and uploaded to the efi-est programme [46], which generates an SSN that was uploaded to Cytoscape for the visualization of the relationships among the peptide sequences. Different settings were tested, and the networks were manually inspected to determine the best settings for generating the SSN (filter type: E-value; filter value: 8). The network was annotated manually according to the predicted AMP classes.

Predicted AMPs were compared, through a local blastp search, against a customized database containing 23 008 already described AMPs. This database was built by incorporating all the AMPs available (on 23 August 2020) in the whole DRAMP database [47] and the APD3 database [48].

## Results

### *Pseudomonas* genome characteristics and annotation

The genome sequences of the selected *Pseudomonas* type strains and the general characteristics of these genomes obtained after their annotation using rast (v2.0) are presented in Data S1 (Table S1). The G+C content of these *Pseudomonas* genomes ranged from 48.26–68.26%. Genomic sizes were comprised of between 3022325 base pairs (bp) and 7 375 852 bp and the number of genes rated ranged between 2913 and 7098.

### Diversity and distribution of biosynthetic gene clusters (BGCs) in *Pseudomonas*

**Fig. 1.**
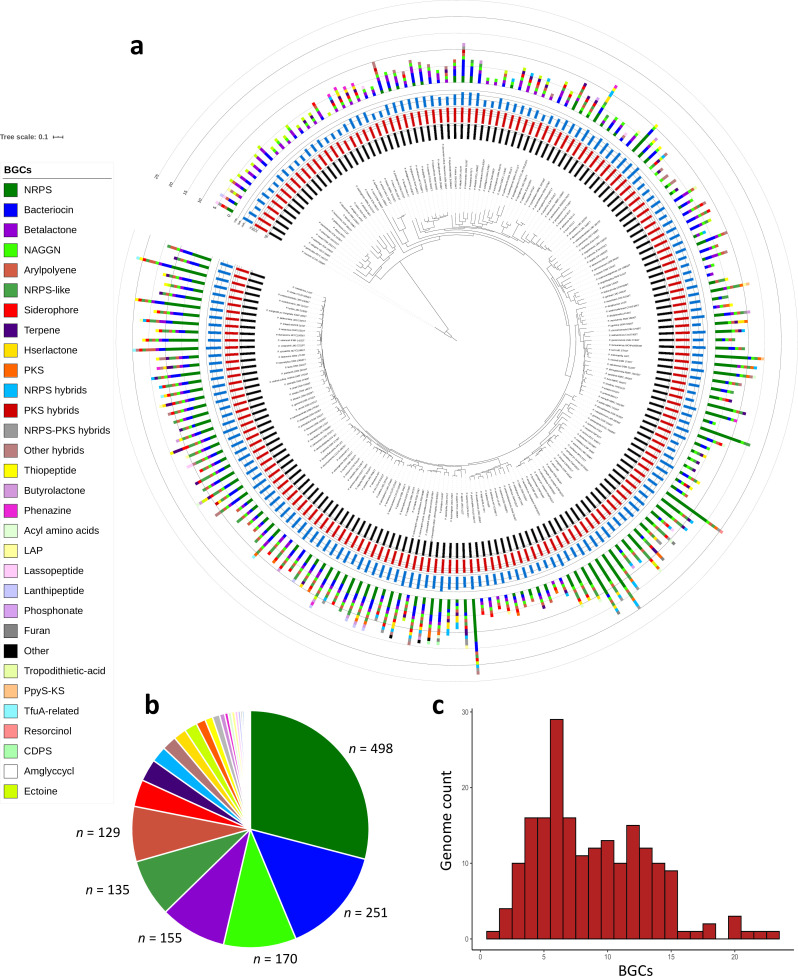
Representation of genome characteristics, BGCs and phylogeny of *Pseudomonas* type strains included in this work. (a) Phylogenetic tree of *Pseudomonas* type strains based on 92 concatenated housekeeping genes. Metadata were drawn with the iTOL programme. The black inner circle represents genome completeness (%); the red circle represents G+C (%) content; the blue circle represents genome length; the outer circle represents BGC types predicted by antiSMASH (colour code reflects each different BGC type). Bar, 0.1 substitution per position. (b) Pie chart summarizing the sum of diverse BGC types within all the genomes of the analyses. The colour code is maintained with the legend at the left. (c) Histogram of the number of *Pseudomonas* type strains (*y* axis) that have different numbers of BGCs (*x* axis).

AntiSMASH was applied for the prediction, annotation and characterization of BGCs related to secondary metabolism. From this analysis, 1721 BGCs were predicted among all genomes, with a distribution of 1–23 BGCs per genome (Fig. 1a, c, Data S1: Table S2, Data S2). Scatterplots show a correlation between the number of predicted BGCs and genome size, but not with the number of contigs (see Fig. S1). Despite this, we observed that some NRPSs (mainly pyoverdine-related NRPSs) are inflated in draft genomes, since some genes of these BGCs are separated into different contigs [49]. *Pseudomonas batumici* UCM B-321^T^ (*n*=23 BGCs), *Pseudomonas extremorientalis* LMG 19695^T^ (*n*=22 BGCs) and *Pseudomonas cichorii* ATCC 10857^T^ (*n*=21 BGCs) were the genomes carrying the highest number of BGCs, while *Pseudomonas caeni* DSM 24390^T^, which had the smallest genome, only contained one. Of these, non-ribosomal peptide synthetases (NRPS) (*n*=498 BGCs) were the most represented type of BGC, followed by bacteriocins (*n*=251 BGCs), N-acetylglutaminylglutamine amides (NAGGNs) (*n*=170 BGCs), ß-lactones (*n*=155 BGCs) and NRPS-like clusters (*n*=135 BGCs) (Fig. 1b). Moreover, 91 hybrids of different clusters were predicted. The distribution of NRPSs (hybrids or not), aryl polyenes, NAGGNs, and PKSs were clade-dependent in the phylogenomic tree. These BGCs were allocated mainly in some clades or absent from others (Fig. 1a and Data S3), whereas the distribution of bacteriocin- or siderophore-related BGCs showed no apparent relation within the *Pseudomonas* taxonomy (Fig. 1a and Data S3).

**Fig. 2.**
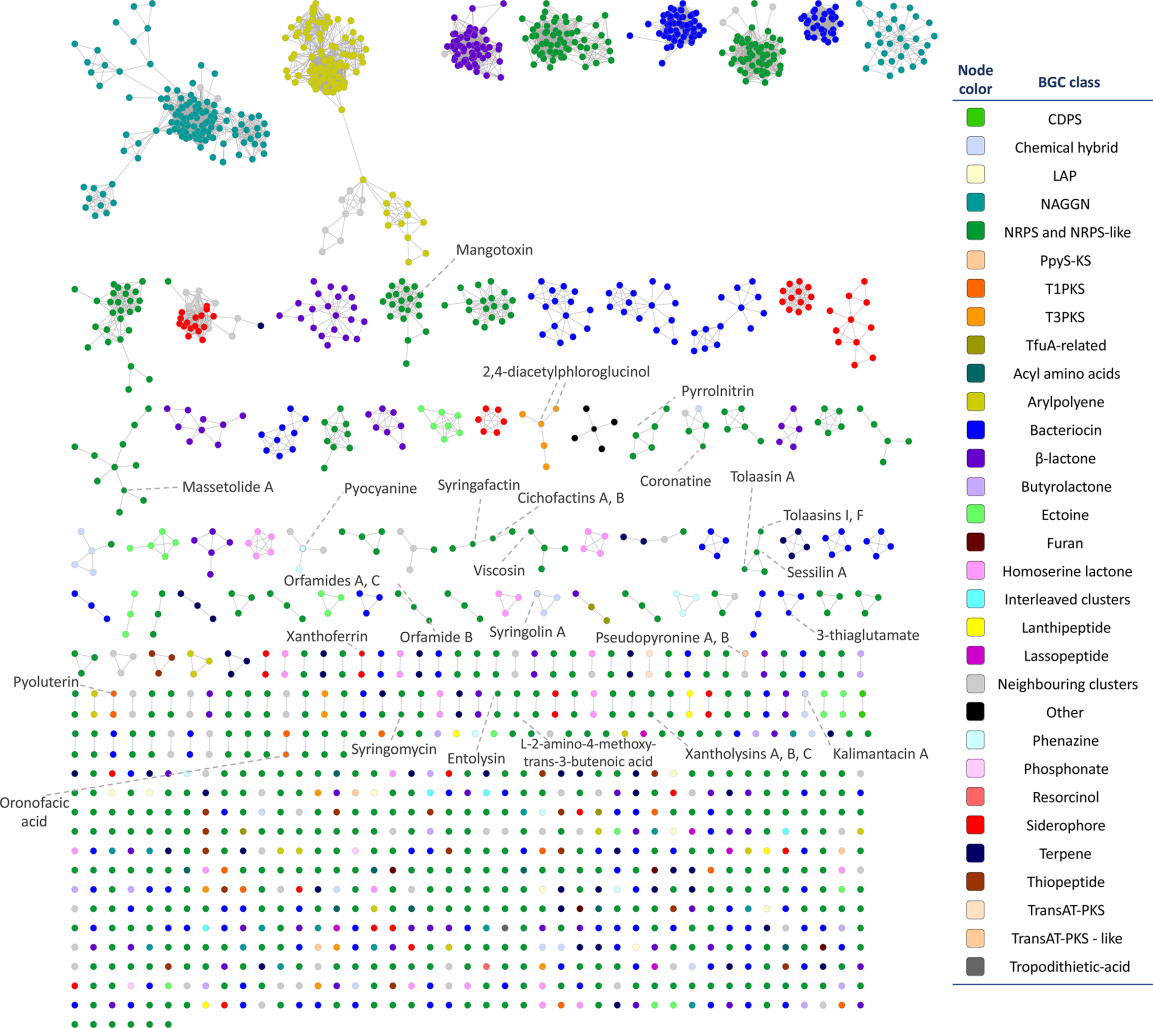
Sequence similarity network (SSN) produced by BiG-SCAPE and visualized and annotated with Cytoscape. Nodes represents BGCs. BGC types are coloured according to the colour legend. MiBIG clusters are labelled according to the metabolic product of the BGC.

The diversity and distribution of these predicted BGCs were assessed through the BiG-SCAPE/CORASON workflow. As a result, 800 GCFs were generated, with an average of two BGCs per family. This analysis included 22 MIBiG reference BGCs, which had similarities to some of the BGCs predicted by antiSMASH. The largest GCF comprised 58 aryl polyene clusters. A clustered heatmap with the presence/absence of the 20 most represented GCFs is included in Supplementary Material (Fig. S2). The construction of SSN based on the similarities between BGCs showed 54 networks (or clans) containing more than two BGCs, of which 19 networks contained more than ten BGCs (Fig. 2a, Data S4). The biggest gene cluster networks do not comprise any MIBiG cluster; therefore, their BGCs represent genetic machinery that may produce novel chemical compounds.

### Co-evolution of *Pseudomonas* species and its secondary metabolism

**Fig. 3.**
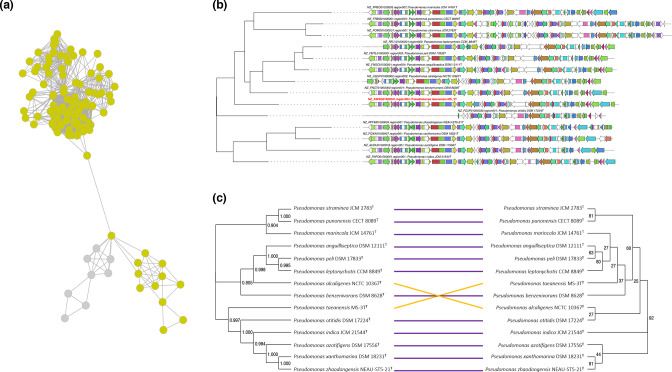
(a) Single network of the BGC SSN representing an example of a clan of GCFs comprising different BGCs of aryl polyenes (mustard nodes) and aryl polyene hybrid clusters (grey nodes). (b) CORASON phylogenetic tree of a GCF of aryl polyenes included in this clan. Each diagram represents a different BGC. The red labelled BGC denotes the representative BGC in the GCF. (c) Tanglegram of the GCF (left; obtained with the BiGSCAPE/Corason workflow) and phylogeny of strains with representatives within the GCF (right; obtained with UBCG tool). Bootstrap values of the phylogeny tree represents the number of UBCG phylogenetic trees that support each differentiation.

The evolution patterns of GCFs comprising more than ten BGCs were studied by constructing tanglegrams of the different GCF trees derived from these analyses and the UBCG phylogenetic trees of strains represented in each of the GCFs (Fig. 3 and Data S5). The tanglegrams included in this analysis cover different types of BGCs such as aryl polyenes, siderophores, NAGGNs, bacteriocins, ß-lactones, NRPSs and NRPS-like clusters. Generally, the clade organization of the BGCs is quite similar or the same as the species clade organization. Indeed, almost all the tanglegrams shows that >85 % (summary table in Data S5) of the node-connections preserves the same evolutionary direction (blue and purple lines in the figures of Data S5). This means that the gene patterns within the BGCs included in the GCFs of *Pseudomonas* evolved in parallel with *Pseudomonas* species evolution gene gains or losses are the main forces driving BGC diversification within a GCF. However, some GCFs do not display differences in gene number but only differences in the amino acid sequence of orthologous proteins.

Similarly, when the different genomes of the analysis are clustered based on the presence/absence of the 800 different GCFs, the different clades from the *Pseudomonas* phylogenomic tree (Fig. 1) show similar species organization than the GCF-distribution based clustering (Fig. S2), although this latter phylogeny does not resolve intra-clade relationships when all genomes in a clade have similar profiles.

### Expansion of genes of secondary metabolites derived from lipid metabolism

**Fig. 4.**
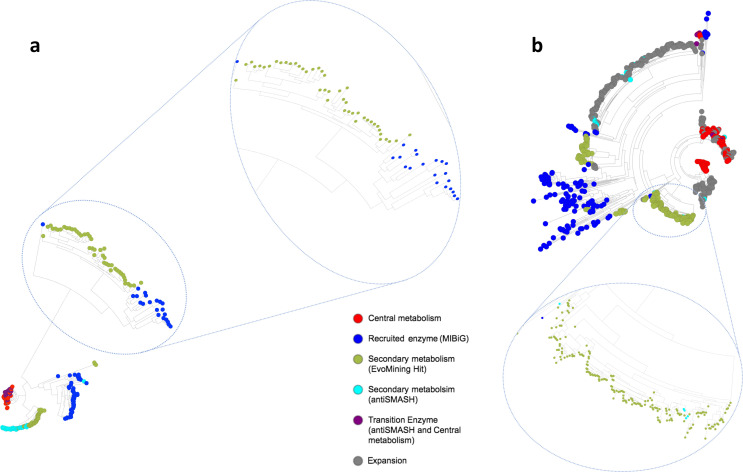
Examples of trees in which the role in the secondary metabolism of proteins related with the lipid metabolism was investigated. Trees were obtained with the EvoMining workflow and visualized with Microreact. (a) Tree no. 13 built with orthologues of a protein with a carboxyl transferase domain. (b) Tree no. eight built with orthologues of an enzyme annotated as an Acyl-CoA dehydrogenase.

Owing to the low number of BGCs detected belonging to the globally distributed PKS cluster type evolved from lipid metabolism (Fig. 1b, Data S1: Table S2, Data S3), we aimed to investigate the possible implication of proteins involved in lipid metabolic pathways in the secondary metabolism of *Pseudomonas *. For this, we employed an evolution-based method for discovering the expansion of genes related to central metabolism that may have taken on functions involved in the production of secondary metabolites. A pan-genome construction allowed us to identify lipid-related metabolic proteins that were widely represented. In total, 20 proteins produced by the *P. aeruginosa* DSM 50071^T^ genome, the type strain for this genus, were selected. Selection criteria were based on the proteins having more than 194 orthologous copies within the pan-genome and being presented in the majority of the genomes. The central metabolism database for carrying out the EvoMining approach (CENTRALlipid_DB; Data S1: Table S3) was comprised of the amino acid sequences of these 20 proteins. The proteins from our genome database were retrieved based on these queries. When the number of orthologous proteins detected for each query was greater than the average number of each protein family per genome, a protein functional expansion was identified. The BGCs predicted by antiSMASH made up the antiSMASH database and these orthologous proteins were also compared to the MIBiG database. Phylogenetic reconstruction for each of the protein families was generated to identify the orthologous copies that may have evolved towards the production of novel metabolites (Fig. 4). Enzyme expansions are depicted in different colours based on their location within the boundaries of BGCs described in the MIBiG database (dark blue) or predicted by antiSMASH (cyan). Enzyme copies that were far from these expansions were presumed to be implicated in central metabolic pathways (red) or were otherwise classified as transition enzymes (purple), when belonging to antiSMASH predictions, but are phylogenetically closer to those of the central metabolism. Thus, enzyme copies that were close to the MIBiG enzymes or antiSMASH hits represented EvoMining hits (green), which may be performing novel functions implicated in new secondary metabolic systems. EvoMining annotated trees are available in Microreact (see Supplementary Material). In addition, many EvoMining hits were observed along with the different trees, suggesting that proteins related to lipid metabolism may interfere in secondary metabolite biosynthetic pathways.

### AMPs prediction and diversity

To gain further knowledge about the specific metabolic potential of *Pseudomonas *, we predicted and classified gene-encoded AMPs using macrel. Short ORFs were predicted and classified based on their chemical composition and their likelihood of being haemolytic or not (Data S6). In sum, 356 cationic AMPs were predicted from 151 out of 194 *Pseudomonas* genomes. Of these, 205 were cationic linear peptides (CLPs) and 151 were disulfide bond-forming peptides (CDPs). Regarding their haemolytic potential, 119 were classified as non-haemolytic (61 CLPs and 58 CDPs) and 237 were classified as being haemolytic (144 CLPs and 93 CDPs). The length of the peptides predicted varied from 11 up to 60 amino acids, and the strains that encoded more AMPs were *P. jinjuensis* JCM 21621^T^ (*n*=9) and *P. saponiphila* DSM 9751^T^ (*n*=9). Local blastp searches using the sequences of all 356 predicted AMPs against our customized database, comprising 23008 AMPs that have already been described (from the DRAMP and APD3 databases), did not yield any significant similarities to any known AMPs. Also, the SSN of the predicted AMPs (Fig. S3a) allowed the analysis of their diversity. It was found that these AMPs were extremely diverse, where only 55 AMPs belonging to networks containing at least two AMPs and 251 AMPs singletons were predicted. Furthermore, some small AMP families having intra-peptide variations that may change the bioactivity of the molecule (Fig. S3b) were identified.

## Discussion

Bacterial and fungal secondary metabolites are a valuable source of antibacterial, antifungal, antiviral and antitumour agents that are being clinically or biotechnologically applied. Consequently, many efforts have been made over recent decades to isolate and characterize novel bioactive compounds that may be used in the fight against human disease [1]. However, microbes contain a vast repertoire of unknown metabolites that has not yet been revealed using traditional culture-dependent techniques. In this sense, genomic-guided approaches are useful in the process of prioritizing and selecting microbial strains and/or genetic machinery that may produce yet to be described metabolites [2]. In this work, we aimed to study the secondary metabolism of the genus *Pseudomonas* based on wide genomic analysis and comparison of their type strains. This selection of representative strains aims to give an overview of the secondary metabolism of the genus. Nonetheless, there may exist genomic divergences within different strains belonging to the same species that imply differences in the secondary metabolome [50]. Thus, considering the huge genetic and functional diversity of *Pseudomonas *, this work does not mean a comprehensive cataloguing of all the metabolic potential of these bacteria.

In this study, we found a large number of BGCs that are unrelated to the BGCs encoding known end products. Also, many of them were found to be clustered together, comprising diverse GCFs that may potentially produce novel chemical compounds. Therefore, research on these BGCs may lead to the discovery of a diverse array of secondary metabolites. Our results show that much of the biosynthetic potential for the genus *Pseudomonas* is associated with NRPSs. NRPSs are mega-enzymes responsible of the modular biosynthesis of peptides with antimicrobial, antitumour, siderophore, surfactant and immunomodulating activities, among others [51]. The abundance of predicted *Pseudomonas* NRPSs is in concordance with the large number of NRPs produced by these bacteria [23]. However, many NRPs are usually predicted with incorrectly established edges when the genomes have not been assembled into a single chromosome [49]. Thus, the number of predicted NRPs may not be accurately represented. Indeed, many predicted NRP clusters, closely related to the one that produces pyoverdine, appear to be systematically separated into different BGCs, with a contig edge located after the first biosynthetic module.

Although the production of bacteriocins by *Pseudomonas* strains has not been extensively documented, this type of BGC makes up the second-largest BGC type found in our analyses, a result that supports the idea of the uncharacterized metabolome encoded within this genus. Indeed, these BGCs appear to be highly diverse, as interpreted from the SSN subnetworks (Fig. 2a). Bacteriocins are ribosomally synthesized antimicrobial peptides, which target diverse microbes [52], and consequently are amenable for being bioengineered, and present an interesting alternative for overcoming multi-drug resistance [53]. Also, the characterization of *Pseudomonas* encoded bacteriocins may help in the fight against antimicrobial resistance.

In general, our results provide strong evidence about how genes related to secondary metabolism are taxonomically classified within certain *Pseudomonas* groups. NRPSs and aryl polyenes were more distributed within a super-clade of *Pseudomonas* than in the rest of the species. NAGGN BGCs are represented along with the entire genus except for a few clades.

Moreover, the evolutionary study of GCF tanglegrams has led us to discover that *Pseudomonas* species (type strains) and BGCs of these GCFs co-evolve together. BGCs of GCFs are placed together in neighbouring branches of the phylogenomic trees. Hence, the phylogenetic prospection of *Pseudomonas* may help in the discoverability of chemical families encoded by these yet uncharacterized GCFs. Similar findings have been described for the genetic machinery of secondary metabolites in *Myxobacteria*; Hoffmann *et al*. [54] described how the presence/absence of chemical families produced by these bacteria are grouped in the same phylogenetic clades. Related research has been carried out on *Streptomyces* strains, where Chevrette and collaborators [14] showed that phylogeny shapes not only the BGC encoded potential, but also metabolomic profiles, and on *Amylocaptosis* species, which also have phylogenetic distribution patterns of secondary metabolites [55]. In contrast, Kautsar *et al*. [56] demonstrated that a relevant percentage of GCFs are not shared among genomes from the same species. Despite the general evolutionary conservation established, we found some minor intersecting lines in GCFs-phylogenomic tanglegrams, which may indicate horizontal gene-transfer events in certain genomes and may drive the chemical diversification of their products [15].

As commented above, the most represented BGC type within the *Pseudomonas* pan-genome was that of the NRPSs, which constitutes a complex module-based genetic machinery for biologically active peptide production. These systems use amino acids derived from the central metabolism as the substrate [57]. Unlike NRPSs, PKSs biosynthesize large secondary metabolites from fatty acid ‘blocks’ instead of amino acids [33]. However, despite PKS being one of the largest distributed types of BGCs in bacteria [26], only a few of them have been predicted in *Pseudomonas* genomes. This trend is also reflected in the MIBIG database, in which there are only 15 PKSs out of the 68 *Pseudomonas* BGCs; this is a low number in comparison with deposited *Streptomyces* BGCs (324/636 PKSs/BGCs). Therefore, we propose that proteins of the lipid metabolism may play broader roles within the secondary metabolism of *Pseudomonas *. According to our results, many orthologues of proteins in the CENTRALlipid-DB were similar to already described proteins within the edges of MIBiG BGCs or in the antiSMASH predictions. These findings are represented as EvoMining hits. Therefore, prospecting into the genetic neighbourhood of these hits may lead to the discovery of novel secondary metabolites or, even, new families of chemical compounds. The reliability of this approach can be exemplified by the discovery of arseno-organic metabolites in streptomycetes by Cruz-Morales and colleagues [12], who based their research on a similar approach. The large number of EvoMining hits derived from our customized databases suggests that some functions of lipid metabolism may have evolved towards the gain of novel functions within the secondary metabolism in *Pseudomonas *.

As mentioned above, genomic-driven strategies are being adopted in natural product research. However, current methods for prospecting the metabolic potential of micro-organisms usually exclude small genes, because of difficulty in accurately detecting and annotating small ORFs (smORFs) [58]. AMPs are short peptides with antimicrobial activity that contain less than 100 amino acids and are encoded by smORFs. Because of their simple genetic machinery, these sorts of peptides can be easily bioengineered and studied. Thus, apart from the work presented here on secondary metabolites encoded by large gene clusters, we also sought to disclose the metabolic potential of *Pseudomonas* strains to produce AMPs based on an *in silico* small peptide search and characterization. A large number of AMPs predicted lacking similarity to already described AMPs in databases unveils an opportunity to discover novel peptides with useful activities. Moreover, the lack of redundancy among AMP within the pan-genome suggests a phylogenetic independent occurrence of AMPs in *Pseudomonas *.

From a species-specific point of view, the genomic comparison of all the *Pseudomonas* type strains included in these analyses allows some to be considered as gifted microbes for the discovery of natural products. Based on the number and diversity of predicted cryptic BGCs and AMPs and the lack of research on their natural products, we suggest that future work on the secondary metabolism of *P. costantinii *, *P. extremorientalis *, *P. batumici *, *P. cichorii* and *P. jinjuensis* is worth considering for the discovery of novel bioactive molecules. However, the possibility that some strains from the same species have different secondary metabolite-related GCFs [56], should be considered.

Finally, we found that many of the BGCs that share high similitude with already described BGCs in *Pseudomonas* have been experimentally validated in the same type strains or even in different strains belonging to the same studied species. For instance, our predictions suggest that *P. protegenes* CHAO^T^ would produce Orfamide A, an insecticidal metabolite discovered from the metabolism of *P. protegens* F6 [59] and in fact, Nguyen *et al*. [24] found through metabolomic analyses that this type strain of this species (CHAO^T^) also produces this compound. Similarly, we detected that the GCF that comprises the BGC described for the synthesis of DAPG is also present in the genomes of the type strains of *P. kilonensis *, *P. brassicacearum* and *P. thivervalensis *. Again, for the type strains of these three species the production of DAPG was experimentally validated [60]. Likewise, it has been reported that strains belonging to the species *P. aeruginosa* [61, 62], *P. entomophila* [63] and *P. syringae* [64] are able to produce phenazines, entolysin and syringolin A, respectively, something that agrees with the results of this study, supporting our predictions.

Our study reveals there is a vast amount of genetic material in *Pseudomonas* that remains undescribed regarding the production of secondary metabolites. Research on this subject may therefore lead to relevant discoveries regarding the potential application of new compounds in clinics and diverse types of industry. Our phylogenomic approaches provide new information about the evolution of natural products and can be used to prioritize BGCs and *Pseudomonas* species for subsequent research. The evolution-based analyses suggest that some of the cryptic secondary metabolites of *Pseudomonas* may be biosynthesized with the involvement of proteins expanded from lipid metabolism.

## Supplementary Data

Supplementary material 1

Supplementary material 2
