# Supplementary material for: Unveiling the genomic potential of Pseudomonas type strains for discovering new natural products
Source: Microb Genom. 2022 Feb 23;8(2):000758. doi: 10.1099/mgen.0.000758 (PMC8942027; doi:10.1099/mgen.0.000758)
Supplement: Supplementary material 1 [file mgen-8-0758-s001.pdf]

Supplementary data

Unveiling the Genomic Potential of *Pseudomonas* type Strains for Discovering New Natural Products

Zaki Saati-Santamaría[a,b*], Nelly Selem-Mojica[c], Ezequiel Peral-Aranega[a,b], Raúl Rivas[a,b,d], Paula García-Fraile[a,b,c].

a. Microbiology and Genetics Department, University of Salamanca, 37007 Salamanca, Spain
b. Institute for Agribiotechnology Research (CIALE), 37185 Salamanca, Spain
c. Evolution of Metabolic Diversity Laboratory, Langebio, Cinvestav-IPN, 36821 Irapuato, Guanajuato, México
d. Associated Research Unit of Plant-Microorganism Interaction, University of Salamanca-IRNASA-CSIC, 37008 Salamanca, Spain

*Corresponding author: Zaki Saati-Santamaría, Email: zakisaati@usal.es

Running title: *Pseudomonas Genomes for Natural Products*

Description of supplementary data

This file contents supplementary data for the main article. However, big tables and special files can be found in Zenodo, under the following link https://doi.org/10.5281/zenodo.4539927 . There you can find the following objects:

- Supplementary Data 1: A spreadsheet with 4 tables: (Table S1) Accession numbers and genome characteristics of the genomes used in this study; (Table S2) summary of BGCs predicted for each of the genomes; (Table S3) proteins used for the conformation of the CentralDatabase for Evomining; (Table S4) AMP predictions.
- Supplementary Data 2: A folder with all the .gbk files for the BGCs predicted by antismash. These files were used as input for BiG-SCAPE.
- Supplementary Data 3: Different trees summarizing the distribution of BGCs in the *Pseudomonas* genus.
- Supplementary Data 4: A file for the visualization of the BGCs-SSN in Cytoscape.
- Supplementary Data 5: Different trees and tanglegrams in which the evolution of GCFs is depicted.
- Supplementary Data 6: A file for the visualization of the AMPs-SSN in Cytoscape.

Evomining trees and metadata have been deposited in Microreact through the following links: (1) https://microreact.org/project/a6j19Z7rVWLQbNkgJjAAkv; (2) https://microreact.org/project/jeB8iymmVuqjocTnitFxvL; (3) https://microreact.org/project/jAXhaH5kiCKCeNfrYiwNVU; (4) https://microreact.org/project/29fHRUP6h5enzX2ruK5eTC; (5) https://microreact.org/project/rR9Dqzg7uFbKsyLsHakG9M; (6) https://microreact.org/project/3S5zZe3NbMoGwHXHfZDbVF; (7) https://microreact.org/project/4CcJmrcRzfTU1NneKYprgd; (8) https://microreact.org/project/2kKgaubCTXpFx7hqGosM4v; (9) https://microreact.org/project/eqWSWgLfS3TZm7eosF34pA; (10) https://microreact.org/project/wtWzxgL2uKbG97FvgFzR2A; (11) https://microreact.org/project/oQvmzS9WrThsAKKFZNzYPZ; (12) https://microreact.org/project/35KUWdaoun3PQMyNeGJuwT; (13)https://microreact.org/project/5gdXTELHJwZYTAfHgiSwBS; (14) https://microreact.org/project/ftwXpDCF15hh91ctCy3YbE; (15)

https://microreact.org/project/vXLU1bGcx5dFZgE9sz6zgL; (16)
https://microreact.org/project/2y4wmrMytAAdnW2irQpbtt; (17)
https://microreact.org/project/bbYpqhxAvyLLaFF6Uy1MH1; (18)
https://microreact.org/project/d8w6z7pbH41R7P5WQ9Y6Dn; (20)
https://microreact.org/project/9iGvZpDzYf3DVVmyBQvn.

In this file there are presented the following supplementary figures for the article:

- **Figure S1**. Scatterplots depicting the number of BGCs/genome and **a)** the number of contigs of the genome or **b)** the size of the genome.
- **Figure S2**. Heatmap of the presence/absence of Gene Cluster Families (GCFs) per genome. **Page 12**
- **Figure S3**. **a)** SSN of Antimicrobial Peptides (AMPs) colored according to the type of AMP and its hemolytic potential. Nodes represents AMPs. **b)** Amino acid sequence alignment of the different AMPs included in the four bigger sub-networks of the AMP SSN. Green shadow in protein multi-alignment represents conservative amino acid positions; red shadow represents a single divergent amino acid.

Figure S1

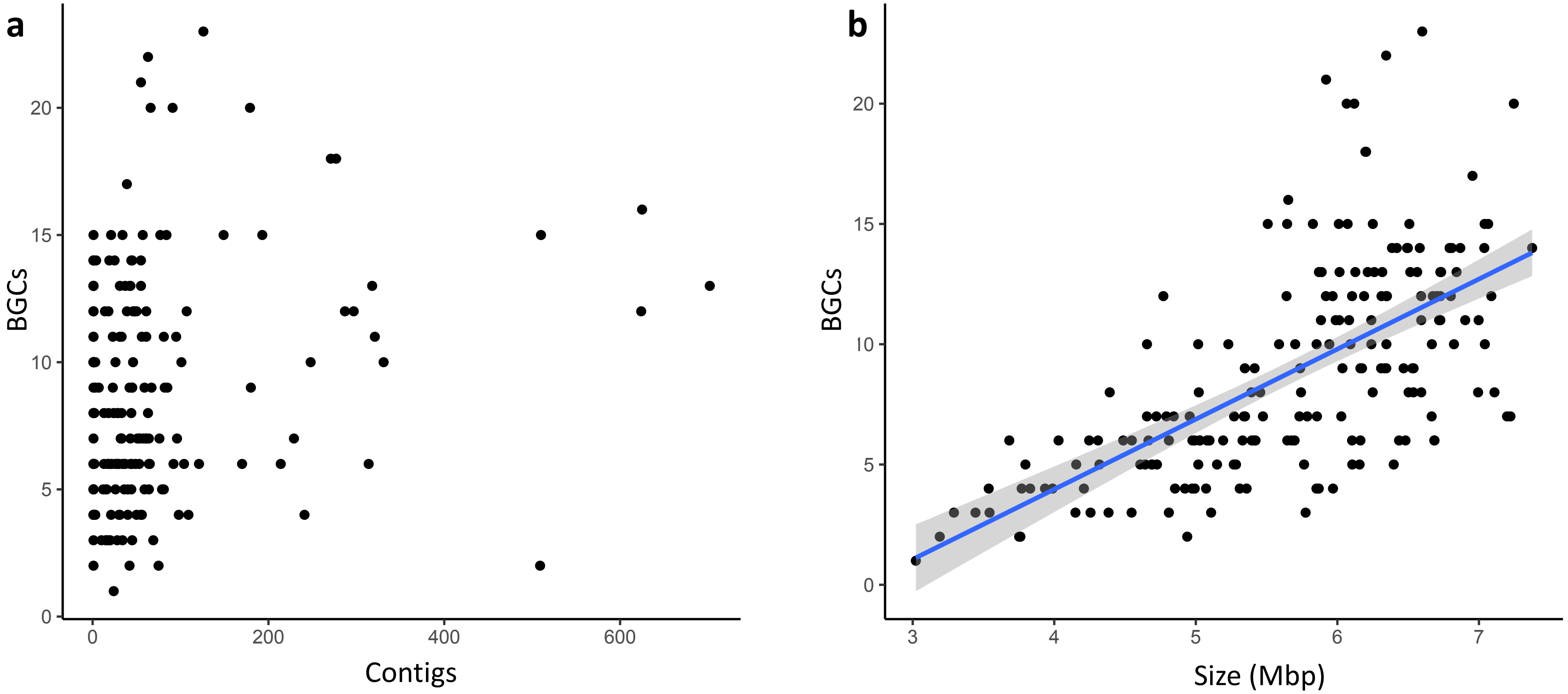
a
BGCs
Contigs
0
5
10
15
20
0
200
400
600
b
BGCs
Size (Mbp)
0
5
10
15
20
3
4
5
6
7

Figure S2

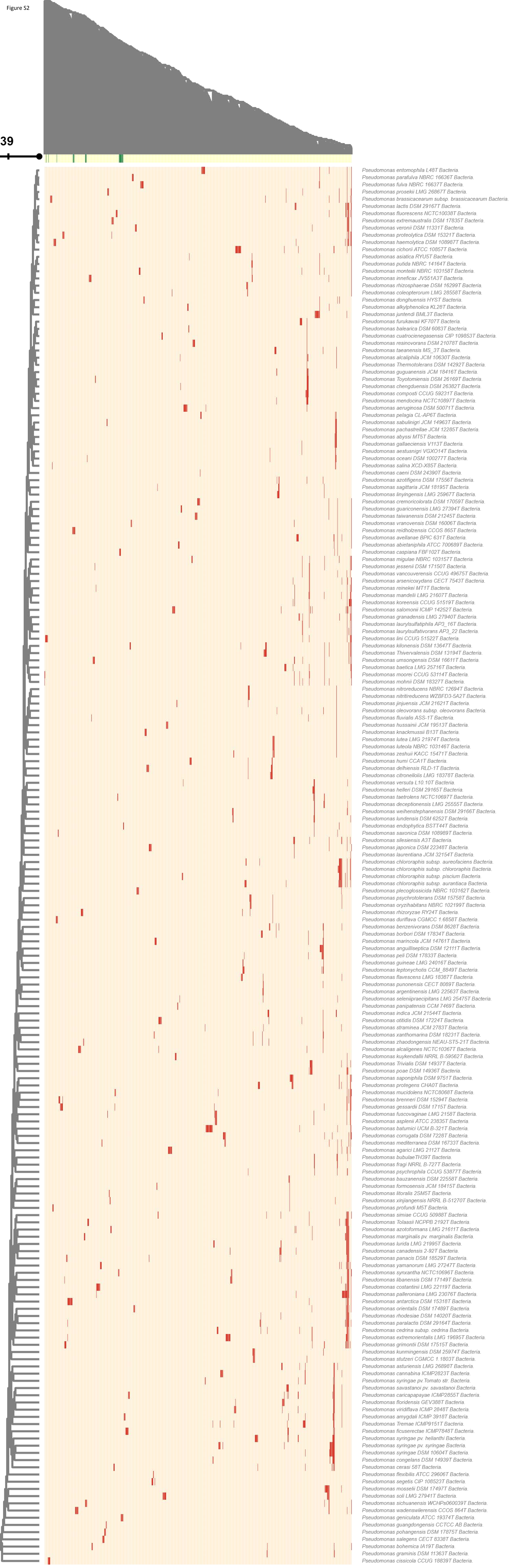
39
Pseudomonas entomophila L48T Bacteria.
Pseudomonas parafulva NBRC 16636T Bacteria.
Pseudomonas fulva NBRC 16637T Bacteria.
Pseudomonas prosekii LMG 26867T Bacteria.
Pseudomonas brassicacearum subsp. brassicacearum Bacteria.
Pseudomonas lactis DSM 29167T Bacteria.
Pseudomonas fluorescens NCTC10038T Bacteria.
Pseudomonas extremaustralis DSM 17835T Bacteria.
Pseudomonas veronii DSM 11331T Bacteria.
Pseudomonas proteolytica DSM 15321T Bacteria.
Pseudomonas haemolytica DSM 108987T Bacteria.
Pseudomonas cichorii ATCC 10857T Bacteria.
Pseudomonas asiatica RYU5T Bacteria.
Pseudomonas putida NBRC 14164T Bacteria.
Pseudomonas monteilii NBRC 103158T Bacteria.
Pseudomonas inefficax JV551A3T Bacteria.
Pseudomonas rhizosphaerae DSM 16299T Bacteria.
Pseudomonas coleopterorum LMG 28558T Bacteria.
Pseudomonas donghuensis HYST Bacteria.
Pseudomonas alkylphenolica KL28T Bacteria.
Pseudomonas juntendi BML3T Bacteria.
Pseudomonas furukawaii KF707T Bacteria.
Pseudomonas balearica DSM 6083T Bacteria.
Pseudomonas cuatrocienegasensis CIP 109853T Bacteria.
Pseudomonas resinovorans DSM 21078T Bacteria.
Pseudomonas taeanensis MS_3T Bacteria.
Pseudomonas alcaliphila JCM 10630T Bacteria.
Pseudomonas Thermotolerans DSM 14292T Bacteria.
Pseudomonas guguanensis JCM 18416T Bacteria.
Pseudomonas Toyotomiensis DSM 26169T Bacteria.
Pseudomonas chengduensis DSM 26382T Bacteria.
Pseudomonas composti CCUG 59231T Bacteria.
Pseudomonas mendocina NCTC10897T Bacteria.
Pseudomonas aeruginosa DSM 50071T Bacteria.
Pseudomonas pelagia CL-AP6T Bacteria.
Pseudomonas sabulinigri JCM 14963T Bacteria.
Pseudomonas pachastrellae JCM 12285T Bacteria.
Pseudomonas abyssi MT5T Bacteria.
Pseudomonas gallaeciensis V113T Bacteria.
Pseudomonas aestusnigri VGXO14T Bacteria.
Pseudomonas oceani DSM 100277T Bacteria.
Pseudomonas salina XCD-X85T Bacteria.
Pseudomonas caeni DSM 24390T Bacteria.
Pseudomonas azotifigens DSM 17556T Bacteria.
Pseudomonas sagittaria JCM 18195T Bacteria.
Pseudomonas linyingensis LMG 25967T Bacteria.
Pseudomonas cremoricolorata DSM 17059T Bacteria.
Pseudomonas guariconensis LMG 27394T Bacteria.
Pseudomonas taiwanensis DSM 21245T Bacteria.
Pseudomonas vranovensis DSM 16006T Bacteria.
Pseudomonas reidholzensis CCOS 865T Bacteria.
Pseudomonas avellanae BPIC 631T Bacteria.
Pseudomonas abietaniphila ATCC 700689T Bacteria.
Pseudomonas caspiana FBF102T Bacteria.
Pseudomonas migulae NBRC 103157T Bacteria.
Pseudomonas jessenii DSM 17150T Bacteria.
Pseudomonas vancouverensis CCUG 49675T Bacteria.
Pseudomonas arsenicoxydans CECT 7543T Bacteria.
Pseudomonas reinekei MT1T Bacteria.
Pseudomonas mandelii LMG 21607T Bacteria.
Pseudomonas koreensis CCUG 51519T Bacteria.
Pseudomonas salomonii ICMP 14252T Bacteria.
Pseudomonas granadensis LMG 27940T Bacteria.
Pseudomonas laurylsulfatiphila AP3_16T Bacteria.
Pseudomonas laurylsulfativorans AP3_22 Bacteria.
Pseudomonas lini CCUG 51522T Bacteria.
Pseudomonas kilonensis DSM 13647T Bacteria.
Pseudomonas Thivervalensis DSM 13194T Bacteria.
Pseudomonas umsongensis DSM 16611T Bacteria.
Pseudomonas baetica LMG 25716T Bacteria.
Pseudomonas moorei CCUG 53114T Bacteria.
Pseudomonas mohnii DSM 18327T Bacteria.
Pseudomonas nitroreducens NBRC 12694T Bacteria.
Pseudomonas nitritireducens WZBFD3-5A2T Bacteria.
Pseudomonas jinjuensis JCM 21621T Bacteria.
Pseudomonas oleovorans subsp. oleovorans Bacteria.
Pseudomonas fluvialis ASS-1T Bacteria.
Pseudomonas hussainii JCM 19513T Bacteria.
Pseudomonas knackmussii B13T Bacteria.
Pseudomonas lutea LMG 21974T Bacteria.
Pseudomonas luteola NBRC 103146T Bacteria.
Pseudomonas zeshuii KACC 15471T Bacteria.
Pseudomonas humi CCA1T Bacteria.
Pseudomonas delhiensis RLD-1T Bacteria.
Pseudomonas citronellolis LMG 18378T Bacteria.
Pseudomonas versuta L10 10T Bacteria.
Pseudomonas helleri DSM 29165T Bacteria.
Pseudomonas taetrolens NCTC10697T Bacteria.
Pseudomonas deceptionensis LMG 25555T Bacteria.
Pseudomonas weihenstephanensis DSM 29166T Bacteria.
Pseudomonas lundensis DSM 6252T Bacteria.
Pseudomonas endophytica BSTT44T Bacteria.
Pseudomonas saxonica DSM 108989T Bacteria.
Pseudomonas silesiensis A3T Bacteria.
Pseudomonas japonica DSM 22348T Bacteria.
Pseudomonas laurentiana JCM 32154T Bacteria.
Pseudomonas chlororaphis subsp. aureofaciens Bacteria.
Pseudomonas chlororaphis subsp. chlororaphis Bacteria.
Pseudomonas chlororaphis subsp. piscium Bacteria.
Pseudomonas chlororaphis subsp. aurantiaca Bacteria.
Pseudomonas plecoglossicida NBRC 103162T Bacteria.
Pseudomonas psychrotolerans DSM 15758T Bacteria.
Pseudomonas oryzihabitans NBRC 102199T Bacteria.
Pseudomonas rhizoryzae RY24T Bacteria.
Pseudomonas duriflava CGMCC 1.6858T Bacteria.
Pseudomonas benzenivorans DSM 8628T Bacteria.
Pseudomonas borbori DSM 17834T Bacteria.
Pseudomonas marincola JCM 14761T Bacteria.
Pseudomonas anguilliseptica DSM 12111T Bacteria.
Pseudomonas peli DSM 17833T Bacteria.
Pseudomonas guineae LMG 24016T Bacteria.
Pseudomonas leptonychotis CCM_8849T Bacteria.
Pseudomonas flavescens LMG 18387T Bacteria.
Pseudomonas punonensis CECT 8089T Bacteria.
Pseudomonas argentinensis LMG 22563T Bacteria.
Pseudomonas seleniipraecipitans LMG 25475T Bacteria.
Pseudomonas panipatensis CCM 7469T Bacteria.
Pseudomonas indica JCM 21544T Bacteria.
Pseudomonas otitidis DSM 17224T Bacteria.
Pseudomonas straminea JCM 2783T Bacteria.
Pseudomonas xanthomarina DSM 18231T Bacteria.
Pseudomonas zhaodongensis NEAU-ST5-21T Bacteria.
Pseudomonas alcaligenes NCTC10367T Bacteria.
Pseudomonas kuykendallii NRRL B-59562T Bacteria.
Pseudomonas Trivialis DSM 14937T Bacteria.
Pseudomonas poae DSM 14936T Bacteria.
Pseudomonas saponiphila DSM 9751T Bacteria.
Pseudomonas protegens CHA0T Bacteria.
Pseudomonas mucidolens NCTC8068T Bacteria.
Pseudomonas brenneri DSM 15294T Bacteria.
Pseudomonas gessardii DSM 17152T Bacteria.
Pseudomonas fuscovaginae LMG 2158T Bacteria.
Pseudomonas asplenii ATCC 23835T Bacteria.
Pseudomonas baturnici UCM B-321T Bacteria.
Pseudomonas corrugata DSM 7228T Bacteria.
Pseudomonas mediterranea DSM 16733T Bacteria.
Pseudomonas agarici LMG 2112T Bacteria.
Pseudomonas bubulaeTH39T Bacteria.
Pseudomonas fragi NRRL B-727T Bacteria.
Pseudomonas psychrophila CCUG 53877T Bacteria.
Pseudomonas bauzanensis DSM 22558T Bacteria.
Pseudomonas formosensis JCM 18415T Bacteria.
Pseudomonas litoralis 2SM5T Bacteria.
Pseudomonas xinjiangensis NRRL B-51270T Bacteria.
Pseudomonas profundi M5T Bacteria.
Pseudomonas simiae CCUG 50988T Bacteria.
Pseudomonas Tolaasii NCPPB 2192T Bacteria.
Pseudomonas azotoformans LMG 21611T Bacteria.
Pseudomonas marginalis pv. marginalis Bacteria.
Pseudomonas lurida LMG 21995T Bacteria.
Pseudomonas canadensis 2-92T Bacteria.
Pseudomonas panacis DSM 18529T Bacteria.
Pseudomonas yamanorum LMG 27247T Bacteria.
Pseudomonas synxantha NCTC10696T Bacteria.
Pseudomonas libanensis DSM 17149T Bacteria.
Pseudomonas costantinii LMG 22119T Bacteria.
Pseudomonas palleroniana LMG 23076T Bacteria.
Pseudomonas antarctica DSM 15318T Bacteria.
Pseudomonas orientalis DSM 17489T Bacteria.
Pseudomonas rhodesiae DSM 14020T Bacteria.
Pseudomonas paralactis DSM 29164T Bacteria.
Pseudomonas cedrina subsp. cedrina Bacteria.
Pseudomonas extremorientalis LMG 19695T Bacteria.
Pseudomonas grimontii DSM 17515T Bacteria.
Pseudomonas kunmingensis DSM 25974T Bacteria.
Pseudomonas stutzeri CGMCC 1.1803T Bacteria.
Pseudomonas asturiensis LMG 26898T Bacteria.
Pseudomonas cannabina ICMP2823T Bacteria.
Pseudomonas syringae pv.Tomato str. Bacteria.
Pseudomonas savastanoi pv. savastanoi Bacteria.
Pseudomonas caricapapayae ICMP2855T Bacteria.
Pseudomonas floridensis GEV388T Bacteria.
Pseudomonas viridiflava ICMP 2848T Bacteria.
Pseudomonas amygdali ICMP 3918T Bacteria.
Pseudomonas Tremae ICMP9151T Bacteria.
Pseudomonas ficuserectae ICMP7848T Bacteria.
Pseudomonas syringae pv. helianthi Bacteria.
Pseudomonas syringae pv. syringae Bacteria.
Pseudomonas syringae DSM 10604T Bacteria.
Pseudomonas congelans DSM 14939T Bacteria.
Pseudomonas cerasi 58T Bacteria.
Pseudomonas flexibilis ATCC 29606T Bacteria.
Pseudomonas segetis CIP 108523T Bacteria.
Pseudomonas mosselii DSM 17497T Bacteria.
Pseudomonas soli LMG 27941T Bacteria.
Pseudomonas sichuanensis WCHPs060039T Bacteria.
Pseudomonas wadenswilerensis CCOS 864T Bacteria.
Pseudomonas geniculata ATCC 19374T Bacteria.
Pseudomonas guangdongensis CCTCC AB Bacteria.
Pseudomonas pohangensis DSM 17875T Bacteria.
Pseudomonas salegens CECT 8338T Bacteria.
Pseudomonas bohemica IA19T Bacteria.
Pseudomonas graminis DSM 11363T Bacteria.
Pseudomonas cissicola CCUG 18839T Bacteria.

Figure S3

a

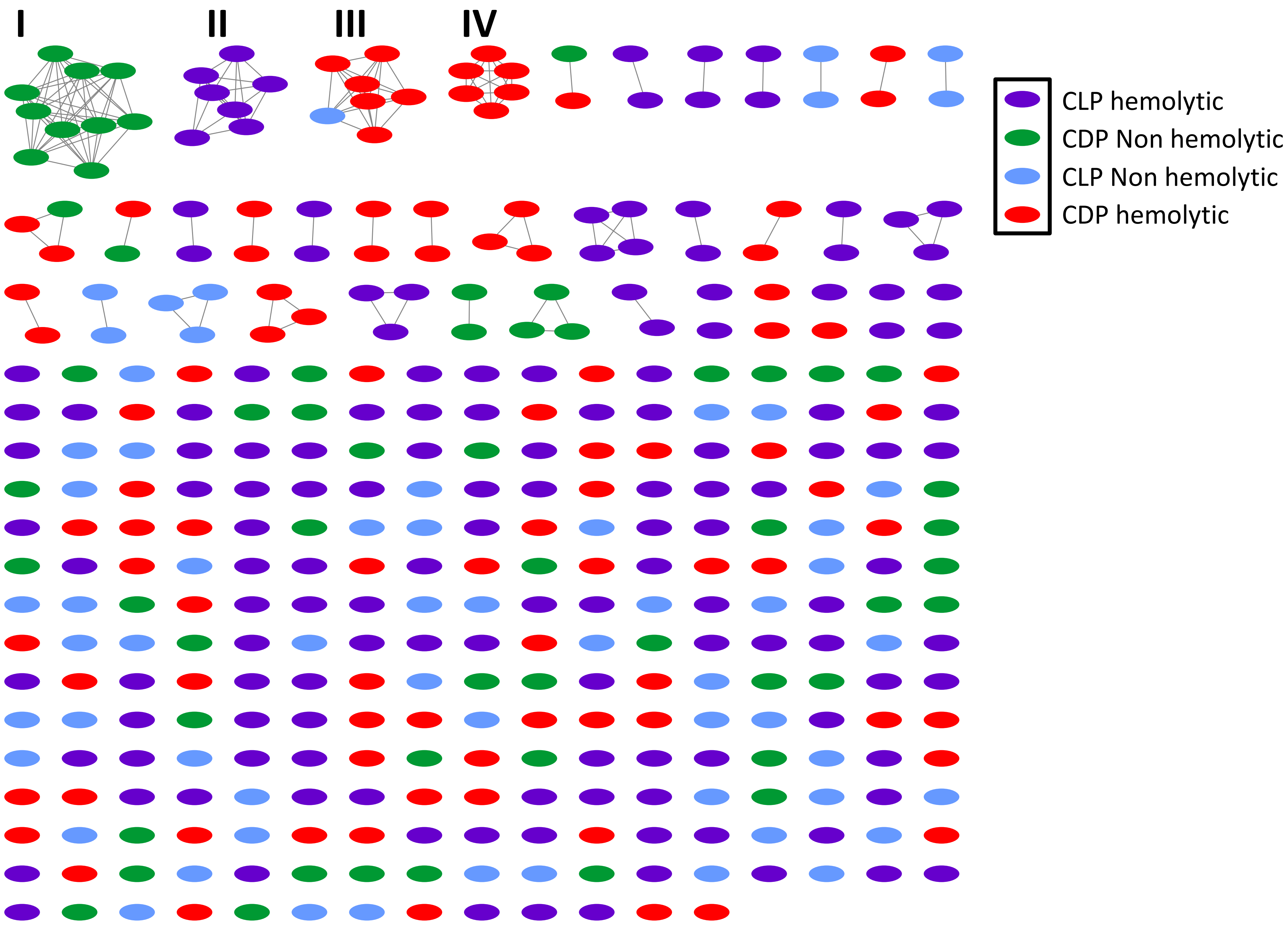

b

I) CAFIDRAKCPGLFACGLRHRPPDAAVKL

II) IATLWFAYVFIYKGPKP
IAPLWFAYVFIYKGPRP

III) PKHTRNHANHTGRTLRPVLAVAGCKAAAGSYFGYWFSH
PKHTRNHANHTGRTLRPVLAVAGCVAPASYYFGYWFSH
SKQTRNHTNHTGRTARPVLAVAGRNAVASFYFGYWFSH
PKHTCNPANHTGRPAHRVLAVAGRNAVAPFYFGYWFSY

IV) ELKLARLRRWLFTLCQARSGQCIK
ELKLARLRRWLFALCQSRSGQCIK
ELKFARLRRWLFALCQARSGQCLKK
ELKLKRLRRWFFALCQSRSGQCLQKP
ELKLKRLRRWLFALCQSRAGQCIRKQ
ELKLKRLRQWLFTLCQARAGQCLKKQ
